# Erratum

**DOI:** 10.1111/jdi.12359

**Published:** 2015-07-02

The authors would like to draw the reader's attention to the error in the following article.

Bhowmik B, Munir SB, Diep LM, Siddiquee T, Habib SH, Samad MA, Khan AKA, Hussain A. Anthropometric indicators of obesity for identifying cardiometabolic risk factors in a rural Bangladeshi population. *J Diabetes Invest* 2013; 4: 361–368.

It was stated that the study was approved by the National Committee for Medical and Health Research Ethics (NEM) of Norway, however it should have been mentioned that it was approved by the Regional Committee for Medical and Health Research (REC) of Norway, instead.

The authors apologize for the error and any confusion it may have caused.

